# Editorial: The Skin Immune Response to Infectious Agents

**DOI:** 10.3389/fimmu.2021.810059

**Published:** 2022-01-12

**Authors:** Fatima Conceição-Silva, Fernanda N. Morgado, Roberta O. Pinheiro, Fabienne Tacchini-Cottier

**Affiliations:** ^1^ Immunoparasitology Laboratory, Oswaldo Cruz Institute, Fundação Oswaldo Cruz, Rio de Janeiro, Brazil; ^2^ Leprosy Laboratory, Oswaldo Cruz Institute, Fundação Oswaldo Cruz, Rio de Janeiro, Brazil; ^3^ Biochemistry Department, University of Lausanne, Lausanne, Switzerland

**Keywords:** skin, immune response, infectious diseases, skin immune system, skin-associated lymphoid tissues (SALT)

This Research Topic highlights different mechanisms associated with the skin immune response against infectious agents. The skin was originally defined as a tissue that covers the body, protecting internal tissues and organs from external physical, chemical, and biological aggressions. A greater understanding of the particularities of the skin immune response began with the identification of skin-associated lymphoid tissues (SALT) (1, 2), and the description of the dermal perivascular units (PVU) comprising layers of CD4 and CD8 T cells around capillaries in the dermis (3). SALT and PVU represent examples of the skin immune response organization (4, 5). Thereafter, the skin immune system (SIS) was defined based on work delineating the presence and function of immune cells at this site (6–10). The identification of SIS, SALT, and other skin immune compartments, such as the immune system of the hair follicle (11), helped to change the definition of the skin from a tissue to a linear organ. Due to its size and total weight (approximately 2m^2^ and about 16% of body weight) it is considered one of the largest organs in the human body. In parallel, the idea of compartmentalization of the immune response with this organ has gained strength *via* the demonstration of decisive events in the control or development of skin diseases and through studies on the *in situ* immune response, particularly for infectious skin diseases. The understanding of the dynamics of immune response to different pathogens that penetrate and multiply in the skin has markedly increased. However, much is still unknown about events related to the encounter between the pathogen and the local immune responses and thus this area still requires further investigation. This is the central idea behind this Research Topic and papers within this collection have assessed the impact of the interaction of SIS with different pathogens.

In this context, the skin immune response during leishmaniasis is described in five articles of this Research Topic. Leishmaniasis is a vector-borne disease caused by protozoans of the genus *Leishmania*, most of which result in tegumentary lesions of affected individuals in endemic areas around the world. The host immune response is considered essential for the progression and control of the disease (12), and different cells and molecules contribute to the inflammatory reaction to *Leishmania* parasites. Although macrophages have been extensively studied, the function of neutrophils is pivotal in the skin immune response against *Leishmania*, as they are rapidly recruited to the infected site. In this context, Passelli et al. review the role of neutrophils in recruiting inflammatory cells to the infected dermis. *Leishmania* spp. are intracellular parasites, mainly residing within mononuclear phagocytic cells, but CD4^+^ and CD8^+^ T-cells play an important role in controlling infection by both cytokine production and direct cytotoxicity. In this context, a new function of CD8^+^ T cells is presented by Koh et al., who demonstrate for the first time the production of lymphocyte extracellular traps (LETs) by CD8^+^ T cells, both *in vitro* and *in vivo* (human tegumentary lesions), suggesting a role of LETs in disease progression. Current therapies targeting programmed cell death (PD)-1 receptor and its ligand (PD-L1) restore T cell activity. In this Research Topic, two articles by Moura et al. and Fonseca-Martins et al. discuss the role of PD-1 and PD-L1 in leishmaniasis. PD-L1 expressed in both macrophages and neutrophils may have suppressive activity and the blockade of PD-L1 may contribute to a reduction in cutaneous pathology. As such, these articles present potential new mechanisms of protection focusing on the PD-1/PD-L1 pathway. Collectively, all the studies mentioned in this topic are relevant to the development of new therapies. Among them, this pathway and so these host-directed therapies represent a promising approach in the treatment of infectious diseases and Novais et al. discuss these perspectives in the field of cutaneous leishmaniasis.

Leprosy is another infection of worldwide importance that affects the skin and nerves. The disease ranges from self-limited to severe forms that can lead to loss of tissue and function when not treated and four manuscripts discuss the skin immune response during leprosy. Although historically the Th1-Th2 dichotomy has been associated with leprosy polarity, an *in vitro* model of *Mycobacterium leprae* infection generated by Leal-Calvo et al. demonstrates that macrophages also have a central role. In addition, the effect of immunosenescence in the skin immune response against the bacilli and the mechanisms in which age-related changes in T cell subsets may influence the onset of leprosy is also discussed by Silva et al. Existence of relapse, resistance to drugs used in the multidrug therapy (MDT), and the low bactericidal activity of rifampicin have previously been described. Even after MDT, multibacillary patients may present high bacillary loads, thus it is important to clarify the mechanisms underlying this phenomenon. In this context, Ferreira et al. demonstrate that a reduction in the bacillary index in the slit-skin smear of patients is associated with higher levels of CXCL10 (IP-10) and IFN-γ and so this. This could be helpful in monitoring the treatment efficacy in leprosy patients (Ferreira et al). Furthermore, CXCL10/IP-10 and IFN-γ are associated with a reduction in the bacillary load by inducing autophagy in host cells. Role of autophagy in the pathogenesis of skin diseases caused by mycobacteria is reviewed by Bittencourt et al. and they discuss the potential for repurposing drugs to target host cells for mycobacterial control.

Other bacteria can produce skin infections and one of the most common is *Staphylococcus aureus*, which can be found as part of the skin’s microbiome, but, in certain circumstances, it can become more aggressive, causing localized or disseminated skin lesions and endogenous disease. Two studies in this collection focus on the relationship between *S. aureus* and the skin immune response. Hamid et al. focus on the neutrophil-*S. aureus* interactions after mouse infection with planktonic or biofilm forms of *S. aureus*. Both bacterial forms induce an early and considerable pro-inflammatory cytokine profile in the lesion together with a predominance of neutrophils. However, some differences in the dynamics of recruitment and functional properties of phagocytes against biofilms are described and the authors discuss their role in promoting an adaptative immune response against *S. aureus*. Another study by Hendriks et al. suggests the importance of CD4^+^ T cells as a barrier to the primary entry site of *S. aureus*.

Another bacterium, *Haemophilus ducreyi*, can induce skin lesions, mainly in children, as well as sexually transmitted genital ulcers in adults. Brothwell et al. review the current literature related to the host-pathogen interaction network, including the adaptation of this organism to its metabolic surroundings and the use of new technologies to better understand *H. ducreyi* pathogenesis.

Many pathogens can colonize and/or invade the skin, producing infections with different grades of severity. In this Research Topic, skin interactions with viruses and fungi are also discussed. Lei et al. review the virus-host immune response interface and discuss both host immune responses and virus immune evasion mechanisms.

Regarding skin immune responses to fungi, different species are able to produce skin infection. Skin mycoses can affect the keratinous layer as well as the epithelial and dermal layers. Depending on the fungus causing the infection, it can produce disease in immunocompetent individuals or hosts with some degree of immunosuppression. One of the most common skin mycoses is caused by a group of fungi known as dermatophytes, which produce a superficial mycosis with a worldwide distribution. In this context, Burstein et al. present an innovative experimental model of dermatophytosis to explore its pathogenesis and further understand the mechanisms of fungal virulence, evasion, as well as immune responses elicited during infection, including the role of C- type lectin receptors and cytokines such as IL-17 and IFN-γ.

*Purpureocillum lilacinum* is considered an emerging pathogen for humans, mainly in immunosuppressed patients, being one of the causal agents of hyalohyphomycosis. Corrêa-Moreira et al. demonstrate a decrease in the number of macrophages and neutrophils as well as in the amount of IL-1β and nitric oxide (NO) in immunosuppressed mice when compared with immunocompetent animals. The authors discuss these results that contribute to a greater understanding of this, as yet, scarcely studied infection.

A plethora of cell types, cytokines, and other molecules are involved in the SIS and the predominance of each one can be stimulated by different pathogens. In this context, Langerhans cells are pivotal to mounting the specific immune response. Oulee et al. identify and evaluate 31 genes that encode proteins that are involved in antimicrobial activity. Based on their results, authors discuss the potential role of Langerhans cells in orchestrating skin immune responses.

The skin is an important organ of the human body, functioning as a homeostatic organ and a mechanical barrier to the external environment, but it is also capable of mounting specific immune responses to different infectious agents such as protozoa, viruses, bacteria, and fungi. The SIS is organized to respond to different stimuli, and disease progression or control can be influenced by the skin immune response (6–10). The articles presented here demonstrate that the skin immune mechanisms related to protection against infectious diseases involve both innate and adaptive immune cells, as well as host characteristics like ageing and metabolic status. A better understanding of the pathways associated with the immunopathogenesis of skin infectious diseases may contribute to the development of new therapeutic and prophylactic strategies. While the present collection provides important new information, many questions remain unanswered. We hope that readers will find this Research Topic a useful reference to understand the complex mechanisms associated with the immune response against pathogens that infect the skin.

## Author Contributions

All authors have written, reviewed, and approved the final version of the manuscript.

## Funding

This work was supported by PAEF-Fiocruz e Programa Jovem Cientista do Nosso Estado - Faperj (E-26/202.760/2019).

## Conflict of Interest

The authors declare that the research was conducted in the absence of any commercial or financial relationships that could be construed as a potential conflict of interest.

## Publisher’s Note

All claims expressed in this article are solely those of the authors and do not necessarily represent those of their affiliated organizations, or those of the publisher, the editors and the reviewers. Any product that may be evaluated in this article, or claim that may be made by its manufacturer, is not guaranteed or endorsed by the publisher.
